# Retraction: Evaluating the Anticancer Activity of Blocking TNF Type 1 Receptors in Thioacetamide-Induced Hepatocellular Carcinoma in a Rat Model

**DOI:** 10.7759/cureus.r225

**Published:** 2026-04-16

**Authors:** Alaa Bagalagel, Reem Diri, Ahmed Noor, Deina Almasri, Hussain Bakhsh, Hussam I Kutbi, Mohammed M Al-Gayyar

**Affiliations:** 1 Clinical Pharmacy, King Abdulaziz University Faculty of Pharmacy, Jeddah, SAU; 2 Clinical Pharmacy, King Abdulaziz University, Jeddah, SAU; 3 Pharmacy, King Abdulaziz University, Jeddah, SAU; 4 Pharmaceutical Chemistry, University of Tabuk Faculty of Pharmacy, Tabuk, SAU; 5 Biochemistry, Mansoura University Faculty of Pharmacy, Mansoura, EGY

The Editor-in-Chief is retracting this article because it contains multiple internal inconsistencies between the reported results, their interpretation, and the stated conclusions, particularly regarding expression of Nrf2 and HO-1. Their expression is described in conflicting directions across different sections, and the findings are not coherently aligned with the proposed mechanisms. These discrepancies raise concerns about the accuracy of data interpretation and the reliability of the study’s conclusions, thereby undermining its overall scientific validity. The authors disagree with the decision to retract.

